# Physical Rehabilitation Core Outcomes In Critical illness (PRACTICE): protocol for development of a core outcome set

**DOI:** 10.1186/s13063-018-2678-4

**Published:** 2018-05-25

**Authors:** Bronwen Connolly, Linda Denehy, Nicholas Hart, Natalie Pattison, Paula Williamson, Bronagh Blackwood

**Affiliations:** 1 Lane Fox Clinical Respiratory Physiology Research Centre, Guy’s and St. Thomas’ NHS Foundation Trust, London, UK; 2 NIHR Biomedical Research Centre at Guy’s and St. Thomas’ NHS Foundation and King’s College London, London, UK; 3 Centre for Human and Applied Physiological Sciences, King’s College London, London, UK; 4 Department of Physiotherapy, Melbourne School of Health Sciences, The University of Melbourne, Parkville, VIC 3010 Australia; 5 School of Health and Social Work, University of Hertfordshire and East & North Hertfordshire NHS Trust, Hertfordshire, UK; 6 MRC North West Hub for Trials Methodology Research, University of Liverpool, Liverpool, UK; 7 Wellcome Wolfson Institute for Experimental Medicine, Queen’s University Belfast, Belfast, Northern Ireland UK; 8 School of Health and Social Work, College Lane Campus, University of Hertfordshire, Hatfield, Hertfordshire AL10 9AB UK; 9 Department of Biostatistics, Institute of Translational Medicine, University of Liverpool, Crown Street, Liverpool, L69 3BX UK; 10 Wellcome Wolfson Institute for Experimental Medicine, Queen’s University Belfast, Belfast, UK

**Keywords:** Physical rehabilitation, Core outcome set, Consensus, Critical illness, Outcome, Measurement

## Abstract

**Background:**

Existing data on physical rehabilitation interventions in critical illness are challenged by outcome heterogeneity that limits data synthesis and translation of research findings into clinical practice. This protocol describes the PRACTICE study to develop a core outcome set (COS) for trials of physical rehabilitation interventions delivered across the continuum of a patient’s recovery from the intensive care unit until reintegration in the community following hospital discharge.

**Methods:**

Mixed methods will be used including: systematic reviews of quantitative and qualitative literature; qualitative interviews with patients and caregivers; a modified Delphi consensus process with researcher, clinician and patient/caregiver stakeholder groups; and consensus meetings for ratification of findings, resolving uncertainty, or developing an action plan for COS implementation.

**Discussion:**

The PRACTICE COS will inform relevant stakeholders about important outcomes regarding physical rehabilitation in critical illness, and may enhance the future design and conduct of trials in this area.

**Trial registration:**

COMET database (www.comet-initiative.org/, Record ID 288, 01/03/13). PROSPERO database (CRD42014008908, CRD42017078549).

**Electronic supplementary material:**

The online version of this article (10.1186/s13063-018-2678-4) contains supplementary material, which is available to authorized users.

## Background

Muscle dysfunction is characteristic in patients following critical illness and is a significant driver underlying persistent physical impairment. Survivors demonstrate deficits in exercise capacity, physical function and health-related quality of life related to physical status for up to 5 years post index intensive care unit (ICU) admission [1–5]. In recent years, the profile of physical rehabilitation in critical illness to mitigate these complications has increased in the UK supported by national guidelines [6, 7]. Furthermore, a growing evidence base of randomised controlled trials (RCTs) has investigated the effectiveness of physical rehabilitation interventions including those delivered in the ICU [8–14], following transfer to the ward [15, 16], post hospital discharge [17–21], and across the recovery pathway [22].

However, one major challenge to the interpretation of existing data is heterogeneity in selection and definition of outcomes used for evaluation. Often trials examining similar interventions measure multiple, dissimilar outcomes. For example, three recent major trials evaluating enhanced exercise-based physical rehabilitation interventions delivered in the ICU measured three [10], seven [11], and 12 [14] outcomes; only one outcome was common across all three studies. The problem is that there is currently no consensus on the most appropriate outcomes for use in these trials [23]. Even the event of consistency in outcome selection, there is often little agreement with regard the measurement tool, timing of assessment, and duration of follow-up; e.g. physical function measured using the Six Minute Walk Test at 6 months [22] or measured using the Short Form-36 Physical Function subscale at 6 weeks post intervention [21]. As evidenced in a recent Cochrane review examining exercise interventions after ICU discharge in survivors of critical illness [24], outcome measure variability impedes meta-analyses of outcomes to evaluate the effect of interventions and thus review evidence cannot recommend guidance for clinical practice.

Establishing a ‘core outcome set’ (COS) is one strategy to address the requirement for outcome transparency in trials. A COS is an agreed, standardised collection of outcomes that would be measured and reported, as a minimum, in all clinical trials for a defined field of interest [25, 26]. Importantly a COS does not preclude researchers from measuring other outcomes of interest relevant to the specific intervention, including the primary outcome. Rather, achieving consensus from key stakeholders on priority outcomes would increase the cumulative value of individual trials for informing evidence-based clinical decision-making. Recent COSs in the critical illness population have focussed on long-term outcomes following hospital discharge in survivors of acute respiratory failure [27, 28], mechanical ventilation [29], and delirium [30]. Outcome selection in complex interventions, such as physical rehabilitation in critical illness, is crucial [31]. At present there is no COS for trials investigating physical rehabilitation interventions at any stage of the recovery pathway for this patient population. The aim of the PRACTICE study, therefore, is to develop a COS for trials of physical rehabilitation interventions delivered across the continuum of recovery from within the ICU to hospital discharge to the community.

## Methods

### Overview

**Fig. 1:**
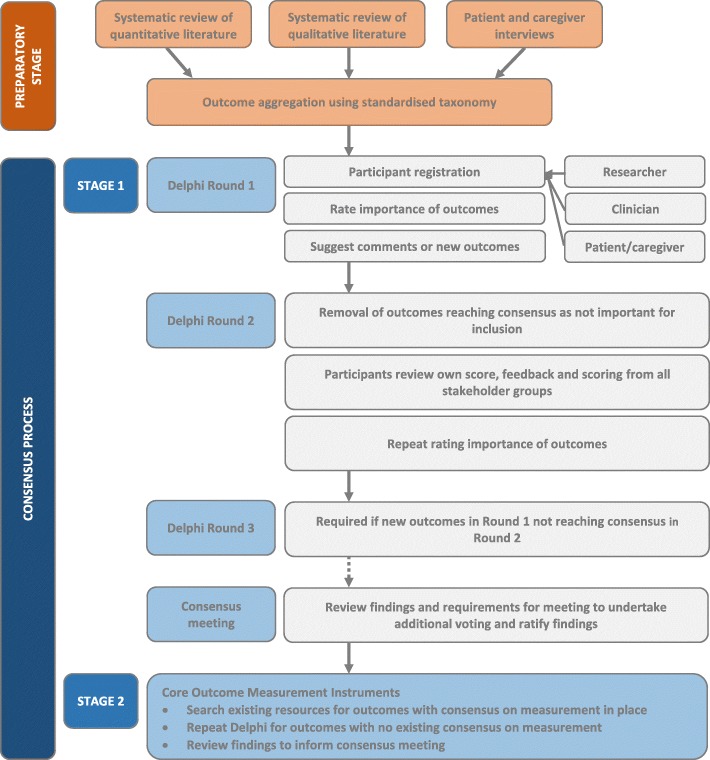
Flow diagram outlining PRACTICE core outcome set development. Stages of consensus for core outcome set and core outcome measurement instruments are summarised. Hashed line (− − ->) indicates specific requirements for a consensus meeting determined on completion of the consensus process

A mixed-methods study adopting the process model for COS development outlined by Williamson et al*.* [25], integrating systematic reviews of quantitative and qualitative literature, qualitative interviews with patients and caregivers (preparatory stage), a consensus process to determine the COS (stage 1), establishing appropriate measurement instruments for the COS (stage 2), and finally dissemination (stage 3) (Fig. 1). This protocol is developed in line with guidance from the Core Outcome Measures in Effectiveness Trials (COMET) Handbook [32], in keeping with methods adopted by prior critical care-related COS [27–30], follows COS-STAD and COS-STAR (Core Outcome Set-Standards for Development [33], and Reporting [34]) recommendations. In addition PRACTICE is supported by the International Forum for Acute Care Trialists (http://www.infactglobal.org/about/).

The Steering Group consists of the study investigators, representing expertise in the field of critical care, physical rehabilitation and COS development; two independent experts with relevant research and clinical expertise; two patient/caregiver representatives; and an independent statistician. We will use the COMET Checklist for Public Research Partners and the COS Study Developers Involved in Designing a COS study Checklist [35] to facilitate engagement with external Steering Group members during the study process. This COS study is registered on the COMET database (http://www.comet-initiative.org/studies/details/288?result=true).

### Scope of the COS

The scope of PRACTICE will specifically apply as follows:

*1. Research or practice setting*

Primarily for adoption in all research trials and clinical studies evaluating physical rehabilitation interventions including, but not restricted to, randomised and controlled trials, comparative study designs with or without concurrent controls, observational cohort studies and case series

*2. Health condition*

Critical illness (life-threatening illness requiring treatment in a high-acuity setting)

*3. Population*

Adult (≥ 18 years of age) patients who have experienced critical illness

*4. Interventions*

Physical rehabilitation interventions include any form of mobilisation, exercise, or adjuncts, such as cycling or electrical muscle stimulation, and delivered across any one or more stages of the continuum of recovery (within the ICU, following ICU discharge, and following hospital discharge to the community)

The PRACTICE study will identify core outcomes for physical rehabilitation interventions delivered at any stage of the recovery continuum, and this requires special consideration. We recognise that different outcome measurement instruments may be more appropriate as a result of patients’ changing clinical status and performance ability as they recover and transition between stages. This may necessitate the use of measurement instruments with more suitable psychometric properties to appropriately reflect that change. We will further understand the extent to which this is evident through data acquired via our systematic reviews of quantitative and qualitative literature, where outcome and outcome measurement instrument data will be categorised according to recovery stage, and the results of the consensus process that identify the core outcomes for inclusion in the set.

### Preparatory stage – information sources

This preparatory stage is vital to inform the selection of outcomes for round 1 of the Delphi consensus. We will conduct (1) systematic reviews of quantitative and qualitative research literature related to physical rehabilitation interventions in critical illness and (2) qualitative, individual, semi-structured interviews with survivors of critical illness and family/caregivers.

#### Systematic review of the trial literature

A full protocol is reported in Additional file 1 and registration (PROSPERO database, CRD42014008908, https://www.crd.york.ac.uk/PROSPERO/). In brief, this review will identify outcomes and associated measurement instruments reported in quantitative studies of physical rehabilitation interventions across the recovery continuum. Data extraction will follow Standard Protocol Items: Recommendations for Interventional Trials (SPIRIT) 2013 recommendations [36], comprehensively characterising outcome reporting, including (1) detail and definition of all primary and secondary outcomes, where reported, (2) specific measurement variables, (3) participant-level analysis metrics, (4) methods of aggregation, and (5) specific time points of measurement. Extracted outcomes will be classified according to a taxonomy for COS development [37] (Table 1), and summarised according to stage of the recovery continuum.

**Table 1:** Abridged details of taxonomy developed for use in core outcome set development

Core area	Domain
Death	Mortality/survival
Physiological or clinical	^a^E.g. cardiac outcomes; nervous system outcomes; respiratory, thoracic and mediastinal outcomes
Life impact	Functioning^b^; global quality of life; perceived health status; delivery of care^c^; personal circumstances
Resource use	Economic; hospital; need for further intervention; societal/carer burden
Adverse events	Adverse events/effect

Abridged from Dodd et al*.* [37]. This taxonomy encompasses 38 domains within 5 core areas. ^a^23 specific domains relating to the underlying cause of affected body system are reported for this core area; ^b^Functioning is expanded to encompass physical, social, role, emotional and cognitive; ^c^Delivery of care includes; for example, acceptability and availability, withdrawal from treatment; process, implementation and service outcomes

#### Systematic review of the qualitative research literature

A full protocol is reported in Additional file 2 and review registration (PROSPERO database, CRD42017078549, https://www.crd.york.ac.uk/PROSPERO/). In brief, this review will identify themes around patient experience of recovery following critical illness across the recovery continuum in relation to receipt of any physical rehabilitation interventions. Patient-reported data that could be considered a potential outcome relevant for the PRACTICE COS will be extracted and mapped to the aforementioned taxonomy [37].

#### Qualitative interviews with patients and caregivers

##### Overview

Meaningful patient and caregiver input into the development of COSs is imperative for highlighting priority outcomes from their perspective that may not be considered by researchers and clinicians [38]. To facilitate this process we will conduct interviews with survivors of critical illness and caregivers to discuss outcomes relevant to physical rehabilitation after critical illness [39].

##### Study population

We will include adult (≥ 18 years of age) survivors of critical illness and caregivers with recollection of the recovery process (including receipt of any physical rehabilitation). For pragmatic purposes, only participants who are fluent in English and UK based will be recruited for interview and we will exclude patients from specialist populations who may have defined rehabilitation pathways in place; e.g. neurological injury or trauma. We will use purposive sampling [40], ensuring maximum variation sampling [41], to reflect a range of age, sex, disease/conditions, and ethnicities. Participants will be identified via patient support groups, charities, patient-focused network groups, snowballing, and personal contacts. Recruitment will continue until data saturation is achieved, defined as no new emergent themes arising from subsequent interviews.

##### Data collection

Semi-structured telephone interviews will be conducted, overseen by an experienced qualitative researcher. Telephone interviews remove the need for an in-person visit either at the participant’s home, hospital, or an alternative location, and enable wider geographical spread of participants. Our interview guide will be developed in conjunction with patient involvement. We will explain the specific purpose of PRACTICE and ask participants what outcomes were of importance, and why, when considering physical rehabilitation in critical illness. Interviews will be audio-recorded for accurate transcription and data analysis.

##### Data analysis

Interview transcripts will be analysed by the research team using thematic analysis [42]. New unique outcomes will be identified, confirmed as relevant and categorised using the aforementioned taxonomy [37].

#### Summarising the information sources

An initial list of outcomes will be generated from the systematic reviews of quantitative and qualitative literature, and qualitative interviews with patients and caregivers. Lay descriptions will be provided, developed with patient/caregiver Steering Group representatives, in addition to any relevant medical terminology. Resources, including plain-language summaries, provided by the COMET Initiative will be used to introduce the Delphi study. Pilot testing will be undertaken prior to commencing the formal Delphi survey.

### Stage 1 – Determining the COS

There is currently no ‘gold standard’ consensus technique to adopt when developing a COS, with a wide variety in methods reported by COS developers [43]. The Delphi technique is increasingly common [44], often with an additional final consensus meeting. Delphi surveys allow for anonymous opinions of participants to be obtained in an objective and neutral manner, such that all responses are given equal weight avoiding influence of perceived values of the views of others, or dominance by stronger individuals [45]. A further advantage of adopting the modified Delphi technique for consensus is that survey rounds can be completed electronically, such that a much larger panel can be included than would be feasible in a face-to-face meeting [45]. For repeated survey rounds, this, therefore, becomes a more cost-effective approach.

#### Participants, sample size, recruitment, and retention

The participant panel will comprise representatives from three key stakeholder groups; ‘clinical researchers’, ‘clinicians’ ,and ‘patients/caregivers’. Optimal panel size to achieve valid consensus in studies using the modified Delphi technique is undetermined [46], influenced by aspects of practicality, analysis time and scope of the question [47]. As large a panel as possible for each stakeholder group will be recruited. Organisations for each stakeholder group will be identified using existing networks of national and international critical care contacts, and web-based searches. Letters of invitation to participate will be emailed to the relevant organisation leads or directly to an individual (where applicable) outlining the study, anticipated timelines for overall commitment and estimated time required for completion of each survey round, and requesting consent to participate.

To minimise attrition bias, which can overestimate degree of consensus in the final results [45, 48], only participants responding favourably to the preliminary invitation to participate will be recruited. During the course of the Delphi survey rounds, strategies will be adopted to facilitate retention of participants including personalised invitations and reminders about survey completion, contact details for the lead researcher, regular checks to verify and update contact details and optimising elements of the online survey including interface, conciseness and speed of completion. A unique identifier will be assigned to each participant to facilitate personalised monitoring of survey completion. The primary means of contact with participants will be via email; however, we will collect a secondary form of contact detail to facilitate sending survey completion reminders.

#### ‘Researcher’ stakeholder group

This group will comprise members from each of the clinical trial group organisations of InFACT, supplemented by senior or corresponding authors from physical rehabilitation publications identified in the systematic review of quantitative literature.

#### ‘Clinician’ stakeholder group

Clinicians will be recruited from international professional organisations relevant to critical care; e.g. physiotherapy, critical care medicine. Clinicians will have a primary role in clinical practice and be medical clinicians at consultant level (or equivalent), and nursing and allied health professionals with at least 3 years’ specialist experience in critical care.

#### ‘Patient/caregiver’ stakeholder group

This group will comprise members of patient and public involvement and engagement groups, patient support groups, charities, or organisations, patient-focused network groups, and personal contacts.

#### Modified Delphi methodology for consensus

Participants will score each outcome according to the Grading of Recommendations Assessment, Development, and Evaluation (GRADE) scale [49], ranging 1–9 in terms of importance for inclusion in the final COS *(1–3, not important for inclusion; 4–6, important but not critical; 7–9, critical for inclusion).* Participants will also be provided with an ‘Unable to score’ response if they consider themselves unable to rate any outcome. Consensus for inclusion by a particular stakeholder group will be defined as ≥ 70% of responses rating the outcome as ‘critical’, i.e. GRADE score of 7 or greater, and less than or equal to 15% of responses rating the outcome ≤ 3 on the GRADE scale, i.e. ‘not important’. Participants will be asked to complete survey rounds within 7 days of receipt; non-respondents will receive reminders for survey completion during an overall survey window of 3 weeks from the original email.

#### Stage 1, Round 1; consensus on core outcomes, i.e. ‘what’ to measure

At the start of Round 1, demographic data (including age, sex, country of residence, duration of clinical and/or research experience, involvement in physical rehabilitation research) will be collected to characterise participants. The Round 1 questionnaire will be structured so that outcomes common to all stages of the recovery continuum will be listed first, followed by outcomes that are specific to individual time points. Within this structure, the order of outcomes will be randomised. For each outcome, participants will rate its importance for inclusion in the PRACTICE COS. Additionally, participants will be able to provide text-based comments and/or additional outcomes for consideration.

#### Stage 1, Rounds 2 and 3

Outcomes meeting consensus for ‘not important for inclusion’ will be removed to maximise efficiency by avoiding re-scoring of redundant outcomes. Remaining outcomes, and new outcomes identified from Round 1, will be carried forward into Round 2. Each participant will be shown their own score from Round 1 and will receive feedback on the average scores from each of the three stakeholder groups [50], and will be asked to re-score the outcome considering this feedback. Participants will have the opportunity to report reasons for any change of score that alters the overall category of importance rating. A third Delphi round will be conducted for new outcomes identified in Round 1 and which do not reach consensus in Round 2, thus allowing for two rounds of rating importance.

#### Consensus meetings

In-person, telephone, or webinar consensus meetings may be held to ratify final COS contents or undertake any additional voting, e.g. in the event that the number of outcomes reaching consensus for inclusion in the COS is perceived to be too many. However there is minimal evidence for the recommended delivery of such meetings [32]. The Steering Group will first review the findings of the consensus process for establishing the COS to determine the requirements for a consensus meeting and its structure, format, and content. Attendees at consensus meetings will comprise a random 10% selection of Delphi participants from each stakeholder group.

#### Nested methodological questions

A number of nested methodological questions will be examined during PRACTICE involving exploratory secondary analyses of the process and dataset. These questions are outlined below, with further details reported in Additional file 3:

1. Which outcomes in the final PRACTICE COS originated from which information sources?
2. How does the final PRACTICE COS feature in existing trials of physical rehabilitation in critical illness?
3. How did different stakeholder groups rate outcomes?

### Stage 2 – Establishing core outcome measurement instruments

When the PRACTICE COS is finalised, we will then determine how to measure the outcomes in the set. We will search existing resources where the outcome measures may have already achieved consensus; e.g. the ImproveLTO (Long Term Outcomes) repository (www.improvelto.com/), and/or other systematic reviews of measurement instruments and published COSs in critical illness. In cases where outcome measures have not already been defined and agreed, we will collate the candidate measurement instruments and undertake a repeat Delphi study using the aforementioned methodology. Participants will be provided with ‘Instrument cards’ to accompany each round in inform decision-making around importance of each outcome measurement instrument. Instrument cards will include descriptions of the instruments, instructions for instrument completion, feasibility of use, psychometric properties for use in the target population and pragmatic items such as cost and licensing requirements. The Instrument cards will be developed in conjunction with www.improvelto.com/ resources, updating existing and generating new cards where necessary. When determining psychometric properties of measurement instruments, we will review existing systematic reviews of relevant articles in the target population and systematically search for any new relevant studies. We will use the COnsensus-based Standards for the selection of health Measurement Instruments (COSMIN) to assess the methodological quality of new evidence [51] in keeping with COSMIN guidelines for selecting outcome measurement instruments in COSs [52]. Where outcomes do not require an instrument measure, definitions will be provided reflecting recommended components [36].

#### Consensus meetings

Findings of the consensus process for determining measurement instruments will be similarly reviewed by the Steering Group for requirements for an additional consensus meeting to ratify findings, or, for example, if there is uncertainty around any outcome measurement instrument. Any consensus meeting will be conducted as described in stage 2.

### Stage 3 - Strategies for dissemination

Multiple formats of dissemination will be employed including peer-reviewed, open access publications, presentation at national and international conferences, engagement with Journal editors, representatives from national and international funding agencies and policy-makers, and summaries (lay and professional versions) for circulation through relevant patient and professional (clinical and research) organisations and networks. In addition we will explore dissemination of study findings via national research infrastructure, healthcare decision-making organisations, e.g. Cochrane, and social media. We will also circulate the final COS via all panel members for their wider dissemination.

### Data management and analysis

All Delphi survey rounds will be delivered electronically using DelphiManager software (COMET Initiative, University of Liverpool, UK). Confidentiality of generated data will be ensured by individual participants’ allocation of unique identifiers, and data storage on secure, encrypted institutional servers. Response rates will be defined as the proportion of recruited participants who completed each survey round, and reported for each stakeholder group. Descriptive statistics will be used to analyse and summarise survey round responses, using GraphPad Prism version 7.0d (GraphPad Software, La Jolla, CA, US, www.graphpad.com).

### Ethics

Individual participants will respond to recruitment advertisements, will be approached directly or will self-volunteer following nomination through their affiliated organization. Participants will receive a specific participant information sheet. Verbal informed consent will be acquired for those participants in the qualitative interviews. For Delphi consensus survey rounds, which will be conducted entirely online, agreement to participate and completion of the surveys will be considered indicative of consent. This study has been approved by the King’s College London BDM (Biomedical Sciences, Medicine, Dentistry, and Natural and Mathematical Sciences) Research Ethics Panel (LRS-17/18–4603), and the UK Health Research Authority National Research Ethics Service North-East Committee (18/NE/0018).

## Discussion

The PRACTICE COS study will follow robust and recommended methodology for its development, including approaches currently adopted by other COS in the critical care specialty to identify core outcomes and their measurement instruments for trials of physical rehabilitation in critical illness. Important stakeholder groups (researcher, clinicians, patients/caregivers) will be included. It is anticipated that adoption of the PRACTICE COS will enhance the design, conduct, and evaluation of future trials in this area.

## Trial status

The systematic reviews of quantitative and qualitative literature have been completed, and recruitment is currently underway for the qualitative interviews. Delphi consensus participants are currently being identified and recruited.

## Additional files

Additional file 1: Protocol for systematic review of quantitative research. (DOCX 23 kb)

Additional file 2: Protocol for systematic review of qualitative research. (DOCX 18 kb)

Additional file 3: Nested methodological questions. (DOCX 13 kb)

## Abbreviations

COMET: Core Outcome Measures in Effectiveness Trials
COS: Core outcome set
COSMIN: COnsensus-based Standards for the selection of health Measurement INstruments
COS-STAD: Core Outcome Set – STAndards for Development
COS-STAR: Core Outcome Set – STAndards for Reporting
GRADE: Grading of Recommendations Assessment, Development, and Evaluation
ICU: Intensive care unit
InFACT: International Forum for Acute Care Trialists
PRACTICE: Physical RehAbilitation Core ouTcomes In Critical illnEss
RCT: Randomised controlled trial
