# Supplementary material for: Physical Rehabilitation Core Outcomes In Critical illness (PRACTICE): protocol for development of a core outcome set
Source: Trials. 2018 May 25;19:294. doi: 10.1186/s13063-018-2678-4 (PMC5970518; doi:10.1186/s13063-018-2678-4)
Supplement: Supplementary file 1 — Protocol for systematic review of quantitative research. (DOCX 23 kb) [file 13063_2018_2678_MOESM1_ESM.docx]

**Physical Rehabilitation Core Outcomes In Critical Illness: PRACTICE, a protocol for a core outcome set development study**

Bronwen Connolly^1, 2, 3, 4^, Linda Denehy^4^, Nicholas Hart^1, 3^, Natalie Pattison^5^, Paula Williamson^6^, Bronagh Blackwood^7^

**Additional File 1 Protocol for systematic review of quantitative literature**

Registration

This review is registered on the PROSPERO database, CRD42014008908 (<https://www.crd.york.ac.uk/PROSPERO/>).

Data sources and search strategy

Four electronic databases (Cumulative Index of Nursing and Allied Health Literature (CINAHL, via EBSCO host), Ovid SP Excerpta Medica Database (EMBASE, 1974 to present), Ovid SP Medline (1946 to present), and Cochrane Central Register of Controlled Trials (CENTRAL)) and two clinical trial registries (<https://clinicaltrials.gov/> and <http://www.isrctn.com/>) will be searched. Search strategies will be reviewed by an experienced information specialist and searches conducted with no temporal or linguistic limitations. Free text and MeSH terms will be used for 'critical care', 'intensive care', 'ICU', 'crit* ill', 'rehabilitation', 'exercise', 'physical function', physiotherapy', 'physical therapy', 'physical activity', 'recovery'. Manual searches of the reference lists of all included articles will be additionally performed.

Eligibility

Initial searches will be screened to remove duplicate and non-relevant material. Retrieved titles and abstracts will be subsequently screened for eligibility independently by two reviewers. Disagreements unable to be resolved by consensus will be arbitrated by a third reviewer where necessary. This process will be repeated for articles retrieved for full text screening.

Included studies, published from 2000 onwards and in the English language, evaluating any physical rehabilitation (PR) intervention delivered at any stage of the recovery continuum, and meeting the following eligibility criteria:

1. *Study design* – prospective quantitative study design including randomised and pseudo-randomised controlled trials, or other comparative study with or without concurrent controls, observational cohort (≥50participants per treatment arm or overall), trial registration and published protocols. Conference abstracts pertaining to eligible studies will be included where no full published data are available (only data on outcomes or measurement instruments will be collected). Case series/studies, retrospective study design, quality improvement, safety, feasibility and physiological response to intervention studies will be excluded.
2. *Participants* - adult patients with critical illness (admission to the ICU, irrespective of causal diagnosis, but requiring invasive mechanical ventilation and multi-organ support)
3. *Interventions* - any PR intervention including early mobilisation, exercise programmes, and adjuncts such as electrical stimulation or cycle ergometry, and delivered across one or more stages of the recovery continuum i.e. within the ICU, post ICU discharge, post hospital discharge. Interventions encompassing respiratory muscle training treatments will be excluded as the focus of this review is rehabilitation primarily targeting peripheral skeletal muscle weakness.
4. *Control/comparator* - any other physical-based intervention, or control or ‘usual’ care

Data extraction

Data extraction will be performed manually and independently by two reviewers using a bespoke, pre-piloted data extraction spreadsheet. Cross-checking of data extraction will be performed by an independent reviewer. Relevant data will include demographic study features, and comprehensive characteristic outcome data adopting the SPIRIT 2013 recommendations [1], including:

1. Detail and definition of all primary and secondary outcomes, where reported
2. Specific measurement variables
3. Participant-level analysis metrics
4. Methods of aggregation
5. Specific time-points of measurement

Outcomes will be classified according to a bespoke taxonomy developed specifically for use during COS development (containing 38 domains of outcomes mapped to 5 core areas) [2], and summarised according to stage of the recovery continuum. Further summary according to type of physical rehabilitation intervention will be conducted if possible. Outcomes common across recovery stages will be highlighted, and the specific measurement instruments used at each stage.

Quality assessment

Risk of bias will not be formally assessed as this will not influence planned data extraction or synthesis which focuses solely on the outcomes and associated metric details reported in the study. Quality of outcome reporting will be assessed using the six-point MOMENT scoring system, where ≥4 is indicative of high quality reporting [3]. The six components (each scoring 1 point) of the MOMENT scale include:

i) Is the primary outcome clearly stated?; ii) Is the primary outcome clearly defined so that another researcher would be able to reproduce its measurement?; iii) Are the secondary outcomes clearly stated?; iv) Are the secondary outcomes clearly defined?; v) Do the authors explain the use of the outcomes they have selected?; vi) Are methods used to enhance the quality of outcome measurement if appropriate?

Data synthesis

Descriptive synthesis of the outcomes used will be conducted. Detail of all outcomes included, primary or secondary status, analysis metric, method of aggregation and timing will be summarised using counts, proportions and ranked order. Frequency of reported individual outcomes and outcome measurement instruments will be determined.

**References**

1. Chan A-W, Tetzlaff JM, Gøtzsche PC, Altman DG, Mann H, Berlin JA, Dickersin K, Hróbjartsson A, Schulz KF, Parulekar WR *et al*: **SPIRIT 2013 explanation and elaboration: guidance for protocols of clinical trials**, vol. 346; 2013.

2. Dodd S, Clarke M, Becker L, Mavergames C, Fish R, Williamson PR: **A taxonomy has been developed for outcomes in medical research to help improve knowledge discovery**. *J Clin Epidemiol* 2017, **Published Ahead of Print**.

3. Harman N, Bruce I, Callery P, Tierney S, Sharif MO, O'Brien K, Williamson P: **MOMENT - Management of Otitis Media with Effusion in Cleft Palate: protocol for a systematic review of the literature and identification of a core outcome set using a Delphi survey**. *Trials* 2013, **14**(1):70.
