# Supplementary material for: Physical Rehabilitation Core Outcomes In Critical illness (PRACTICE): protocol for development of a core outcome set
Source: Trials. 2018 May 25;19:294. doi: 10.1186/s13063-018-2678-4 (PMC5970518; doi:10.1186/s13063-018-2678-4)
Supplement: Supplementary file 2 — Protocol for systematic review of qualitative research. (DOCX 18 kb) [file 13063_2018_2678_MOESM2_ESM.docx]

**Physical Rehabilitation Core Outcomes In Critical Illness: PRACTICE, a protocol for a core outcome set development study**

Bronwen Connolly^1, 2, 3, 4^, Linda Denehy^4^, Nicholas Hart^1, 3^, Natalie Pattison^5^, Paula Williamson^6^, Bronagh Blackwood^7^

**Additional File 2 Protocol for systematic review of qualitative research**

Registration

This review is registered on the PROSPERO database, CRD42017078549 (<https://www.crd.york.ac.uk/PROSPERO/>).

Data sources and search strategy

Five electronic databases (Cumulative Index of Nursing and Allied Health Literature (CINAHL, via EBSCO host), Ovid SP Excerpta Medica Database (EMBASE, 1974 to present), Ovid SP Medline (1946 to present), Cochrane Central Register of Controlled Trials (CENTRAL), and PsycInfo (1806 to present)) will be searched. Search strategies will be developed and refined with an experienced information specialist, followed Peer Review of Electronic Search Strategies recommendations [1, 2], and conducted with no temporal or linguistic limitations. Free text and MeSH terms will be used for 'critical care', 'intensive care', 'ICU', 'crit* ill', 'rehabilitation', 'exercise', 'physical function', physiotherapy', 'physical therapy', 'physical activity', 'recovery', 'qualitative', 'patient reported experience measure*', 'content analysis', 'thematic analysis', ‘phenomenology', 'grounded theory', 'narrative', 'ethnography', 'focus group*', 'interview*', and 'experience'. Manual searches of the reference lists of all included articles will be additionally performed.

Eligibility

Initial searches will be screened to remove duplicate and non-relevant material. Retrieved titles and abstracts will be subsequently screened for eligibility independently by two reviewers. Disagreements unable to be resolved by consensus will be arbitrated by a third reviewer where necessary. This process will be repeated for articles retrieved for full text screening.

Included studies, published from 2000 onwards, reporting the patient experience following critical illness at any stage of the patient recovery pathway, with the following eligibility criteria:

1. *Study design* - All qualitative methodologies (excluding auto-ethnographies of n=1) will be included. Editorials, opinion pieces or similar narrative commentaries, and all non-English-language papers (for reasons of practicality) will be excluded.
2. *Participants* - adult (≥18years of age) patients with critical illness (admission to the ICU for 48hours or more) or their caregivers. Specialised patient populations e.g. neurological, burns or trauma injury will be excluded as existing specific rehabilitation pathways may be in operation for these cohorts.
3. *Exposure* – experience of critical illness recovery, with or without receipt of any physical rehabilitation intervention, at any stage of the recovery continuum
4. *Control/comparator* – no comparator/control is applicable for this review

Data extraction

Data extraction will be performed manually and independently by two reviewers using a bespoke, pre-piloted data extraction spreadsheet. Cross-checking of data extraction will be performed by an independent reviewer. Broadly data extraction will encompass an overview of the theoretical framework adopted by the study, data collection methods, analysis approaches, and major themes reported around experience from critical illness across the recovery continuum (including or excluding receipt of physical rehabilitation). Any patient-reported data that could be considered a potential outcome, or measurement instrument, relevant for the PRACTICE COS will be additionally extracted and mapped to the aforementioned taxonomy [3].

Quality assessment

Two reviewers will independently assess individual studies for quality using a framework based on recommendations from the Cochrane collaboration (<http://methods.cochrane.org/qi/supplemental-handbook-guidance>) and Critical Appraisal Skills Programme (<http://www.casp-uk.net/casp-tools-checklists>). Studies will not be excluded based on quality, but the quality of studies will be reported. A third reviewer will be used in the event of any disagreements between the first two reviewers

Data synthesis

A narrative synthesis is planned, after using thematic analysis to describe the themes from the studies. A meta-synthesis will be performed pending sufficient appropriate data. Findings for patients who have participated in physical rehabilitation as part of their recovery from critical illness will be explored as a subgroup.

**References**

1. McGowan J, Sampson M, Salzwedel DM, Cogo E, Foerster V, Lefebvre C: **PRESS Peer Review of Electronic Search Strategies: 2015 Guideline Statement**. *J Clin Epidemiol* 2016, **75**:40-46.

2. Sampson M, McGowan J, Cogo E, Grimshaw J, Moher D, Lefebvre C: **An evidence-based practice guideline for the peer review of electronic search strategies**. *J Clin Epidemiol* 2009, **62**(9):944-952.

3. Dodd S, Clarke M, Becker L, Mavergames C, Fish R, Williamson PR: **A taxonomy has been developed for outcomes in medical research to help improve knowledge discovery**. *J Clin Epidemiol* 2017, **Published Ahead of Print**.
