# Supplementary material for: Physical Rehabilitation Core Outcomes In Critical illness (PRACTICE): protocol for development of a core outcome set
Source: Trials. 2018 May 25;19:294. doi: 10.1186/s13063-018-2678-4 (PMC5970518; doi:10.1186/s13063-018-2678-4)
Supplement: Supplementary file 3 — Nested methodological questions. (DOCX 13 kb) [file 13063_2018_2678_MOESM3_ESM.docx]

**Physical Rehabilitation Core Outcomes In Critical Illness: PRACTICE, a protocol for a core outcome set development study**

Bronwen Connolly^1, 2, 3, 4^, Linda Denehy^4^, Nicholas Hart^1, 3^, Natalie Pattison^5^, Paula Williamson^6^, Bronagh Blackwood^7^

**Additional file 3. Nested methodological questions**

A number of nested methodological questions will be examined during PRACTICE involving secondary analyses of the process and dataset. These questions are as follows;

1. Which outcomes in the final PRACTICE core outcome set originated from which information sources?

*Aims*

To explore if all information sources were necessary to identify the outcomes contributing to the final core outcome set, and what similarities and differences existed between outcomes identified from quantitative and qualitative sources.

*Method*

Outcomes reaching consensus for inclusion in the PRACTICE core outcome set will be compared to the outcomes identified from the original systematic reviews of quantitative and qualitative literature, and qualitative interviews with patients/caregivers. Outcomes derived from quantitative and qualitative sources will be compared for common and unique outcomes.

*Analysis*

Descriptive reporting of which, and how many, outcomes originated from which source, and commonalities and differences between sources.

1. How does the final PRACTICE core outcome set feature in existing trials of physical rehabilitation in critical illness?

*Aim*

To characterise how existing trials of physical rehabilitation interventions in critical illness may have adopted the PRACTICE COS.

*Method*

Outcomes used in randomised controlled trials of physical rehabilitation interventions included in the systematic review of quantitative literature will be compared to the final PRACTICE COS set.

*Analysis*

Descriptive reporting of the number and frequency with which the final PRACTICE COS outcomes feature in existing randomised controlled trials of physical rehabilitation in critical illness.

1. How did different stakeholder groups rate outcomes?

*Aim*

To explore similarities and differences between stakeholder groups’ rating of outcomes?

*Method*

The rating of outcomes during the development of the final PRACTICE COS will be reviewed across the three stakeholder groups (researchers; clinicians; patients/caregivers).

*Analysis*

Descriptive reporting of the rating of individual outcomes by each stakeholder group.
